# CMR in Heart Failure

**DOI:** 10.4061/2011/739157

**Published:** 2011-08-16

**Authors:** Daniel M. Sado, Jonathan M. Hasleton, Anna S. Herrey, James C. Moon

**Affiliations:** The Heart Hospital, University College London Hospitals NHS Trust, 16-18 Westmoreland Street, London W1G 8PH, UK

## Abstract

Heart Failure (HF) is a common syndrome with multiple causes. Cardiovascular magnetic resonance (CMR) is a medical imaging technique with significant advantages, allowing the understanding of aetiology and pathophysiology of HF in the individual patient, permitting specific therapy to be administered and predicting prognosis. This paper discusses the diverse role of CMR in HF.

## 1. Introduction

Heart failure (HF) is a syndrome arising from any cardiac disease severe enough to impair the heart's ability to support the physiological circulation. Clinically, it is characterised by a number of symptoms and signs most of which relate to fluid accumulation; particularly fatigue, breathlessness, cough, and peripheral oedema. The incidence and mortality of HF is high with more than 24,000 deaths a year in the UK [1] and a 30% mortality within 1 year of first hospital admission [2]. In the United States, 1 in 8.6 death certificates (282,754 deaths/year) mention HF [3]. Management of HF involves prevention, diagnosis of the underlying aetiology, treatment of symptoms, and prevention of disease progression. This may include drug therapy aimed at maladaptive processes within the pathophysiology of heart failure—typically blockade of the renin-angiotensin system, drugs for symptoms (for example diuretics) and nonpharmacological treatment (particularly exercise and dietary changes). Device therapy may also be appropriate (ICD, biventricular pacemaker, ventricular assist devices) and at end stage, cardiac transplantation may be considered. Specific therapy tailored to the etiology of HF is also available. Common causes of HF are coronary artery disease (mainly infarction but with some role for myocardial ischaemia), hypertensive heart disease, valvular heart disease, diabetes, myocarditis, cardiomyopathies, tachycardia-induced cardiomyopathy, and systemic diseases with cardiac manifestations. Within an individual, often more than one etiology is present. Common contributory factors exacerbating symptoms within individuals may include lung disease, obesity, detraining, and simultaneous disease in virtually any other organ system.

The approach to the HF work-up is systematised. Diagnosis of the syndrome is based on history, clinical examination, electrocardiography, chest radiograph, and blood tests (including BNP and tests for heart failure mimics—hypoalbuminaemia, hypothyroidism, and anaemia). Imaging in HF is challenging and requires a multimodality approach. Echocardiography is the first-line investigation for assessment of cardiac anatomy, function, and dynamics. It is cheap, widely available and can be done at the bedside. However, it is limited by poor image quality in some patients and gives very little information about tissue characteristics and coronary anatomy. Cardiovascular magnetic resonance (CMR) provides additional incremental data in patients with HF, providing accurate quantification of systolic dysfunction, determining aetiology and prognosis and predicting response to and selecting therapy. The excellent image quality obtained by CMR allows a detailed examination of cardiac structure and function, without the need for ionising radiation. There are also the key advantages of tissue characterisation (unique to CMR) and perfusion imaging. Evaluation of HF is therefore one of the major current roles of CMR in clinical medicine with the pilot EuroCMR registry of 11,000 patients finding that the most common reason for referral for CMR was to evaluate for potential myocarditis/cardiomyopathy [4]. In this registry, CMR impacted on the management of two thirds of patients and in 16% a new cardiac diagnosis was made that had not been apparent previously. 

Assessment of the coronary arteries is currently limited to assessment of origins and proximal courses using CMR. The small calibre of the vessels and movement during the cardiac cycle make flow limiting disease difficult to evaluate using this modality [5]. Cardiac CT or percutaneous angiography are therefore required for anatomical assessment of nearly all patients with potential coronary disease. 

This paper provides an overview of the principles of CMR and then discusses its role in specific conditions that can cause HF.

## 2. The CMR Process

CMR is the use of MRI to image the heart. Any modern MRI scanner can be used, once ECG gating and specific sequences for cardiac work are installed. Scans are typically performed during multiple 7–15 second breath holds, with standardized protocols according to the referral indication [6, 7]. The first 10 minutes of a CMR study is always to assess anatomy and cardiac structure and function—a volume and function study. This typically uses double-oblique imaging orientated to the long and short axes of the heart using steady-state free precession (SSFP) ECG gated cine imaging. The result is a highly reproducible, stereotyped set of images which are acquired in the same way in all CMR centres [8]. At the end of almost any CMR study is late gadolinium enhancement imaging (LGE, see below) looking for myocardial scar from infarction, cardiomyopathy, or myocarditis. In between, additional techniques may be used, including myocardial blood velocities/flow (e.g., for detecting shunts, valve disease), cardiac iron quantification (iron overload), oedema imaging (acute myocarditis, sarcoidosis, infarction), T1-weighted imaging (pericardial disease and masses), tagging (pericardial disease, diastology), real-time imaging during respiration (diaphragm paralysis, pericardial constriction), stress imaging (vasodilator or inotropic stress for ischaemia/hibernation), tumour tissue characterisation, and early postcontrast imaging (thrombus, microvascular obstruction). A typical HF protocol would take 40 minutes. For further details, see Table 1.

## 3. Gadolinium Enhancement Imaging

CMR is excellent for distinguishing different soft tissues based on their magnetic properties described by the relaxations times T1, T2, and T2*. Normal and abnormal myocardium may have different relaxation times, but the addition of a gadolinium contrast agent greatly magnifies these differences. Gadolinium is chelated and behaves as a passive, extravascular, extracellular contrast agent. After an intravenous bolus, three time phases are considered. The first pass can be used for perfusion imaging and is often given during vasodilator stress to detect ischaemia. Early after contrast, severely hypovascular regions will not enhance (thrombus or microvascular obstruction in acute myocardial infarction). In the late phase (5 minutes plus post bolus), contrast lingers in areas of infarction or focal fibrosis due to slower contrast kinetics and a greater volume of distribution in extracellular water associated with collagen. When imaged using a sensitive sequence (inversion recovery), focal fibrosis appears bright—a region of late gadolinium enhancement (LGE). This can be assessed either visually or quantitatively based on relative enhancement compared to the background [9]. The extent and pattern of LGE varies according to the underlying disease process and is frequently of prognostic significance and can be diagnostic of the underlying etiology.

## 4. CMR Safety

**Figure 1:**
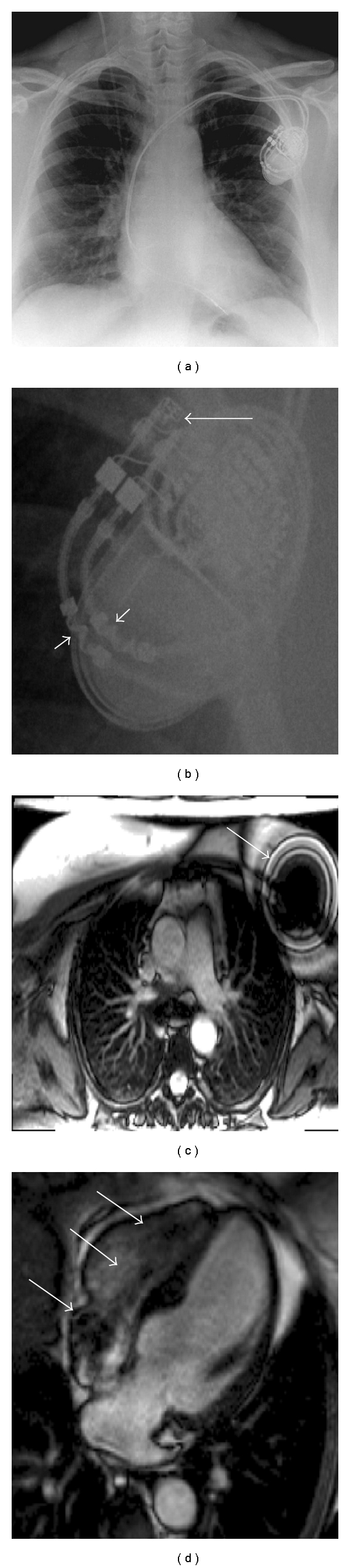
A CMR conditional pacemaker. (a, b) show the pacemaker on a chest X-ray. On (b) the arrows point to MRI conditional markers on the header and leads. (c) shows a large artifact from the pacemaker box on a transverse white blood stack. (d) shows suseptibility artifact from the pacemaker leads in the heart.

Sternal wires and all cardiac prosthetic valves and cardiac stents in use in the UK are CMR safe at 1.5 and 3 T. Many intravascular clips are not safe. All ICDs and most pacemakers are not safe with the risk of malfunction and significant heating of the pacemaker leads. There are now CMR conditional pacemakers (generator and leads) available (Figure 1). In the vast majority of patients, gadolinium contrast agents are safe—safer than iodine-based contrast. However, in patients with an estimated glomerular filtration rate of <30 mL/min, only cyclic gadolinium-based contrast agents should be used, and then only after risk-benefit analysis and at the minimum dose because of the possibility of the severe condition, nephrogenic systemic fibrosis (NSF) [10].

## 5. Practical Clinical Issues

The most common reason for the failure to complete a CMR examination is claustrophobia. In one study, this resulted in 4.2% of patients refusing to undergo CMR [11]. However, with scanning patients feet first, prone, the occasional use of low dose oral (or intra venous) sedation and sensitive treatment, nearly all patients who arrive in a department can be successfully scanned. Some patients with HF may be difficult to scan—either due to inability to lie flat, poor breath holding, or atrial fibrillation with uncontrolled ventricular rates. Real-time, ungated cine imaging, parallel imaging, highly efficient sequence, arrhythmia rejection sequences, and single shot late enhancement sequences help overcome some of these difficulties. On occasions, scanning may be deferred or repeated when patients clinically improve, particularly if the patient is not be able to lie flat due to an acute decompensation of their HF.

## 6. CMR in Myocardial Infarction

### 6.1. Acute Myocardial Infarction

CMR can be safely carried out in patients following acute infarction and primary angioplasty and aids risk stratification [12–15]. T2-weighted imaging detects myocardial oedema, allows for early diagnosis of myocardial ischaemia, area at risk and salvage [16], and may differentiate between acute and chronic infarction. T2* changes occur in intramyocardial haemorrhage in acute infarction, an adverse prognostic marker [17–20]. Microvascular obstruction, the tissue equivalent of the no-reflow phenomenon, can be easily detected by CMR. It is associated with adverse ventricular remodelling and clinical outcome [21, 22]. Within areas of microvascular obstruction, myocardial haemorrhage may occur which carries an incremental adverse prognosis [23].

Following the onset of coronary occlusion, irreversible myocardial injury/myocyte necrosis develops as a transmural wavefront which begins in the subendocardium and moves progressively towards the epicardium [24]. LGE CMR can detect even small volumes of focal injury (in the order of grams) and identifies subendocardial scar not otherwise captured by regional contractile function or SPECT imaging [25]. The volume of LGE in acute infarction has been shown to be a strong independent predictor of both heart failure and poor outcomes [26].

The excellent spatial resolution and wide field of view in CMR also allows for identification of the complications of acute infarction including thrombus, true or false aneurysm, papillary muscle involvement, and ventricular septal defect.

### 6.2. Chronic Myocardial Infarction (Figure 2)

The use of CMR in chronic myocardial infarction helps to guide therapeutic and revascularisation decisions. As well as looking at rest perfusion images, imaging can be performed during vasodilator stress to derive flow reserve. Perfusion defects can therefore be observed and the cause of these can be clarified by comparing the LGE images and regional wall motion abnormalities on cine imaging. Low dose dobutamine stress CMR can also be used to assess myocardial contractile reserve. 

Left ventricular dysfunction secondary to coronary artery disease typically is either reversible as in states of ischaemia, or potentially irreversible, as in transmural myocardial infarction. As ischaemic cardiomyopathy is potentially reversible, the assessment of myocardial viability is extremely valuable. CMR can assess multiple markers of myocardial viability in a single examination [27]. 

Myocardial infarction has a characteristic LGE pattern due to the wavefront of myocardial necrosis. This always involves the subendocardium at the core of the infarct, although it may be transmural. Transmurality predicts potential functional recovery but with a grey-zone of 50% transmural infarction recovery with revascularisation it is more difficult to predict [28]. Low dose dobutamine can provide incremental information. 

The identification of viable (dark on late gadolinium imaging) myocardium in a patient with regional or global left ventricular systolic dysfunction in the setting of ischaemic heart disease can represent hibernation, stunning, dilated cardiomyopathy, or dyssynchrony [28–30]. Such myocardium may recover functionally with time (stunning), revascularisation (hibernation), or resynchronisation (see below). In addition, medical therapy (beta-blockers in particular) are more likely to improve cardiac function in the presence of living but dysfunctional myocardium.

## 7. CMR in Inherited Cardiomyopathy (Figures 3 and 4)

### 7.1. Hypertrophic Cardiomyopathy (HCM)

This autosomal dominant disease resulting in otherwise unexplained left ventricular hypertrophy can present with a number of different clinical phenotypes and in some patients will result in the development of HF and sudden death [31]. CMR is an important investigation in its work-up. High resolution cine images allow accurate hypertrophy assessment, particularly useful in apical hypertrophy which is difficult to visualise using echocardiography [32]. Complex left or right ventricular outflow tract obstruction is well visualised to plan intervention. First pass gadolinium imaging can show perfusion defects whilst LGE sequences show the quantity and distribution of fibrosis, which predicts future HF [33]. In contrast to ischaemic cardiomyopathy, the late enhancement pattern will not follow a coronary distribution and rarely involves the subendocardium. CMR can also identify phenocopies (or genocopies) of HCM, with characteristic findings in Anderson Fabry disease (such as basal inferolateral mid wall late enhancement) and amyloidosis (different gadolinium kinetics with global subendocardial enhancement), both of which may have specific therapeutic options [34].

### 7.2. Familial Dilated Cardiomyopathy (FDCM)

FDCM is characterised by left ventricular (LV) dilatation and impairment on the background of a family history [35]. CMR provides the accurate assessment of LV and right ventricular (RV) volumes, mass, and systolic function. Around 35% of patients with FDCM will have mid myocardial wall LGE, with some evidence that this is a risk factor for sudden cardiac death and ventricular arrhythmia [36]. The pattern of LGE can sometimes be useful in suggesting the underlying genetic basis of FDCM, with dystrophinopathies often showing enhancement of the basal inferolateral segment [37]. It can also help to rule out phenocopies of FDCM, particularly ischaemic cardiomyopathy in older patients.

### 7.3. Arrhythmogenic Right Ventricular Cardiomyopathy (ARVC)

This disease is characterised by fibrofatty infiltration of the right or both ventricles [38]. It may present with sudden cardiac death, although in latter stages can cause biventricular systolic impairment and hence HF. It is a challenging disease to diagnose as assessment of the RV by echocardiography is difficult and limited due to its complex and variable geometry. The multiple image planes that can be performed during CMR make RV assessment less unreliable. Recently published international task force guidelines emphasise assessment of regional wall motion abnormality and RV volumes and ejection fraction [39]. Although not mentioned in the task force criteria, CMR can also be used to identify areas of myocardial fibrofatty infiltration [40].

### 7.4. Left Ventricular Noncompaction (LVNC)

This disease is characterised by the presence of a thick endocardium with prominent trabeculation and deep recesses communicating with the LV cavity [41]. In some cases it can result in HF via LV systolic impairment. Noncontrast-enhanced echocardiography is limited in the evaluation of LVNC as the LV apex is often poorly visualised. Cine CMR imaging will provide excellent views of the LV apical endocardium, with early and late gadolinium contrast imaging allowing evaluation for thrombus. However at present LVNC is a complicated and often debateable diagnosis particularly due to wide normal variation and there is no consensus on diagnostic criteria [42].

## 8. CMR in Intracardiac Shunting (Figure 5) and Other Congenital Heart Disease

Heart failure is the final common pathway for many types of congenital heart disease. CMR can assist in the morphological diagnosis, functional assessment, angiography, flow and tissue characterisation, monitoring ventricular function, and tracking change over time. Flow analysis allows shunt detection—a ratio of >1.5 is considered significant and likely to result in patient symptoms (including those of heart failure) at some stage [43]. Cine imaging can then be used to demonstrate the shunt anatomy including ASDs, VSDs, anomalous pulmonary venous drainage, and more complex disease [44]. CMR is the gold standard for investigating complex disease, and its use has dramatically reduced the need for invasive angiographic assessment [45]. Its primary limitation is that direct measurement of pressures cannot be made and so pulmonary vascular resistance cannot be directly assessed.

## 9. CMR in Pericardial Disease

Pericardial disease, particularly pericardial restriction can cause HF in the presence of preserved ejection fraction but impairing cardiac filling [46]. CMR can detect the anatomy of pericardial thickening; the local and systemic functional consequences (e.g., tethering of the pericardium to myocardium, effusions), and the nonquantitative haemodynamics of pericardial constriction with ventricular: ventricular interaction either at rest or on real-time cine imaging during inspiration (where preferential filling of one ventricle due to respiration restricts the other, with a clear septal deviation associated) [47–49]. The assessment of pericardial calcification is better performed using CT scanning.

## 10. CMR in Valvular Heart Disease

Echocardiography is the first-line test for valvular heart disease, providing excellent assessment of haemodynamics and anatomy in most patients. CMR has incremental value, particularly where echocardiographic windows are poor [50]. 

Cine imaging will allow precise evaluation of ventricular size and function which can be important in deciding when to surgically intervene in valvular disease [51]. It will also provide high-quality imaging of the valve anatomy allowing accurate planimetry of the aortic valve to be performed. In aortic valve disease, cine imaging and gadolinium enhanced aortography will show the anatomy of the aorta in three dimensions. CMR is also very useful for assessment of pulmonary valve disease which can be difficult with echocardiography.

Flow sequences will allow the calculation of the regurgitant fraction in regurgitant valve disease and the forward flow maximal velocity in stenotic disease [52, 53]. 

## 11. CMR in Specific Acquired Cardiomyopathies (Figure 6)

### 11.1. Sarcoidosis

In the thorax, sarcoidosis usually manifests with disease in the lungs, lymph nodes, or heart. Autopsy evidence has shown that cardiac involvement can occur in up to 30% of cases [54]. CMR assessment can show extracardiac disease. If cardiac involvement is present, T2-weighted sequences can highlight areas of acute inflammation. LGE sequences will show enhancement in areas of disease, which is usually multifocal and in the mid or subepicardial layers [55]. In patients who require endomyocardial biopsy to make the diagnosis, there is some evidence that targeting areas with LGE increases the diagnostic yield of the procedure [56].

### 11.2. Myocarditis

This condition is caused by inflammation of the myocardium, most commonly as result of infection [57]. It can progress and result in a dilated cardiomyopathy and hence HF [58]. T2-weighted CMR sequences will show areas of acute inflammation. LGE will identify areas of necrosis or severe oedema. This is most commonly seen in the septal or basal inferolateral wall in the mid or subepicardial layers [59]. Myocarditis is a common finding on CMR in patients presenting with chest pain and an ECG/biomarkers suggesting myocardial infarction, but a normal coronary angiogram. One recent study found that 60% of patients presenting in this manner had myocarditis [60]. Such patients may not have any regional wall motion abnormality and so tissue characterisation using CMR is the only noninvasive method for making the diagnosis.

### 11.3. Iron Overload

The most common cause of iron overload cardiomyopathy is repeated blood transfusions in patients with beta thalassemia major. This results in a 50% mortality in patients under the age of 35 years [61]. CMR allows the accurate quantification of cardiac iron levels using the T2 star method [17]. This allows identification of patients who are at risk of developing HF, allowing more aggressive iron chelation therapy to be administered.

### 11.4. Cardiac Amyloidosis

Cardiac involvement is common in patients with AL or TTR amyloidosis. The presence of amyloid protein results in accelerated removal of gadolinium from the blood and increased myocardial uptake, such that more of it is present in the myocardium than the blood pool [62]. This change in gadolinium kinetic behaviour is almost unique to amyloidosis. The LGE pattern in amyloidosis is often subendocardial and seen in a circular pattern throughout the LV. This is in keeping with histological evidence demonstrating where cardiac amyloid deposition occurs [63].

### 11.5. Eosinophilic Diseases

Cardiac hypereosinophilia (e.g., malignant, Loefflers; Churgg-Strauss) causes endomyocardial fibrosis, valve disease and papillary muscle dysfunction, diastolic dysfunction, intracardiac thrombus formation, and heart failure. These are highly characteristic and well diagnosed and followed by CMR using T2-weighted imaging, early and late gadolinium techniques [64, 65].

### 11.6. Chagas Disease

Endemic in South America, the chronic phase may result in heart failure [66] characterised by myocardial fibrosis, sometimes mimicking infarction. CMR LGE is common and the extent of LGE correlates with disease severity [67].

## 12. CMR and Biventricular Pacing

Cardiac resynchronisation therapy (CRT) using biventricular pacing is a commonly used treatment for patients with HF and limiting symptom [68]. Upto 1/3 of patients fail to respond despite stratification by the ECG QRS duration. Results using echocardiographic markers of dyssynchrony have been mixed [69]. CMR cine and tagging sequences are being investigated [70], but one promising marker is ventricular scar burden and distribution. A high scar burden, predicts nonresponse, and even more robustly, posterior scar (the location of the LV lead) virtually precludes response [71]. It may also be possible by CMR to determine coronary sinus LV branch anatomy to target the LV lead away from scar areas [72].

## 13. Conclusions

The incidence of HF remains high throughout the world. CMR using the methods described above, is establishing itself as the gold standard non invasive method for phenotyping this condition, providing important insight into both the aetiology and pathophysiology of this syndrome in individual patients. In future years we expect that CMR will provide us with continuing improvements in image quality. Many new sequences are likely to become available which for example will improve LGE imaging [73] and allow assessment of diffuse myocardial fibrosis [74]; a variable that at present can only be assessed by invasive biopsy. Most importantly, we envisage CMR becoming more widely available, allowing a greater number of patients with HF to undergo this important investigation.

## Figures and Tables

**Figure 2:**
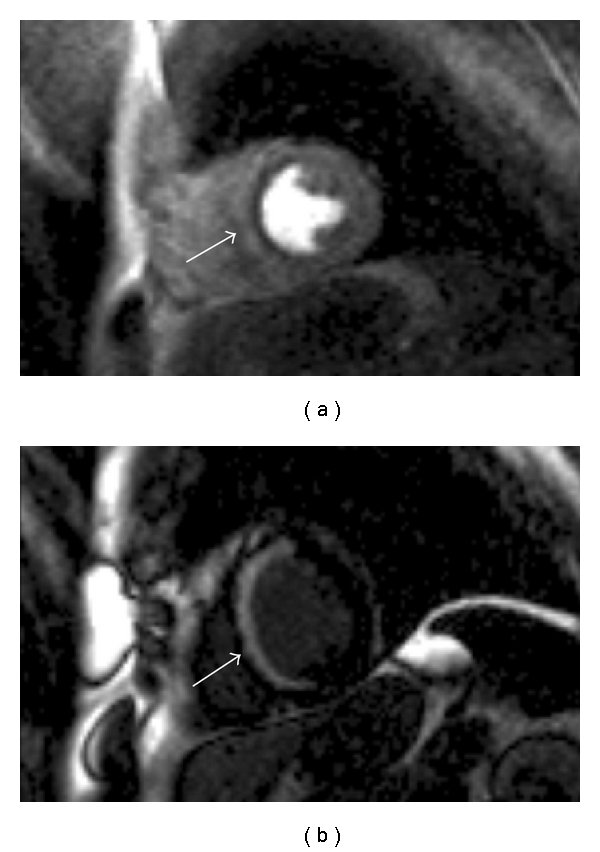
CMR in a patient with ischaemic cardiomyopathy. The cine imaging had shown normal wall thickness throughout but septal akinesis. (a) First pass perfusion sequence following administration of vasodilator stress with adenosine. A defect is seen throughout the septum. (b) Almost full thickness LGE is seen in the septum highly suggestive that this area is nonviable.

**Figure 3:**
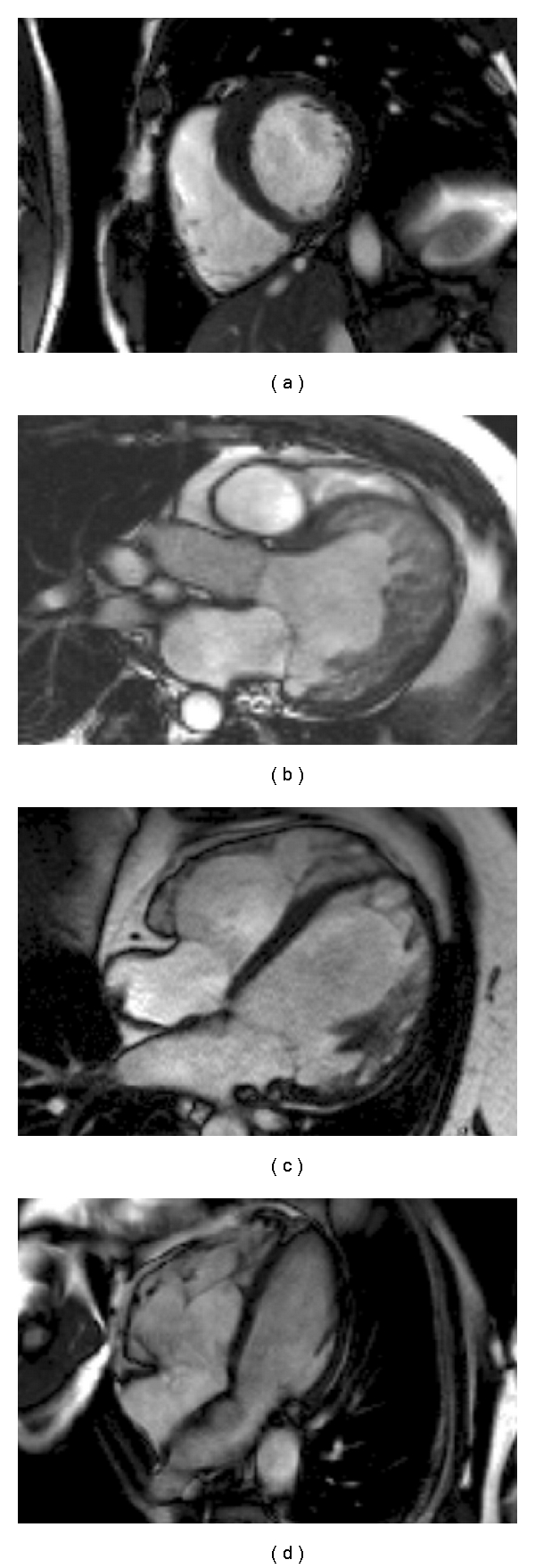
CMR cine sequences demonstrating the differing morphologies of inherited cardiomyopathies. (a) HCM: asymmetric basal anterospetal wall hypertrophy; (b) LVNC: left ventricular hypertrabeculation and dilatation; (c) FDCM: left ventricular wall thinning and dilatation; (d) ARVC: right ventricular dilatation.

**Figure 4:**
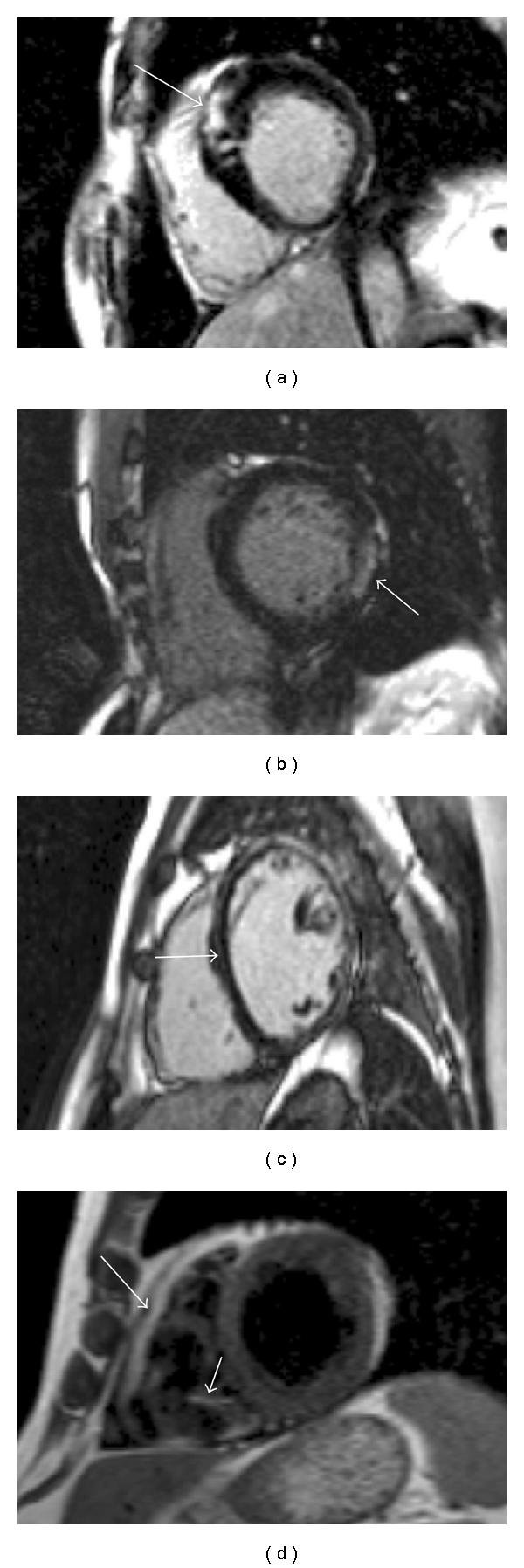
Tissue characterisation in inherited cardiomyopathy. (a) Patchy anteroseptal wall LGE in HCM; (b) Basal inferolateral wall LGE in Anderson Fabry Disease; (c) Septal and anterior mid wall LGE in familial FDCM; (d) RV fatty replacement in ARVC on the trabeculae and possibly the free wall.

**Figure 5:**
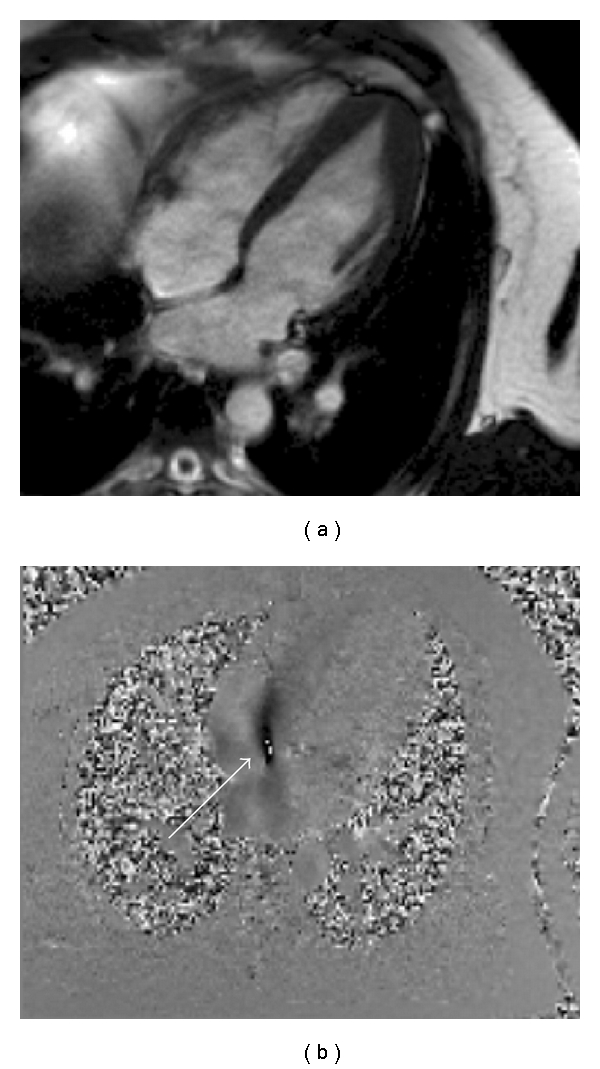
A patient with known apical HCM in whom transthoracic echocardiography had suggested a secundum ASD. (a) Cine 4 chamber image showing apical hypertrophy (b) flow sequence planned using the 4 chamber view shows flow across the intra atrial septum from left to right (arrowed), confirming the diagnosis. In this case, although the right heart does not appear dilated, the Qp : Qs was calculated as 2 showing the presence of a significant shunt.

**Figure 6:**
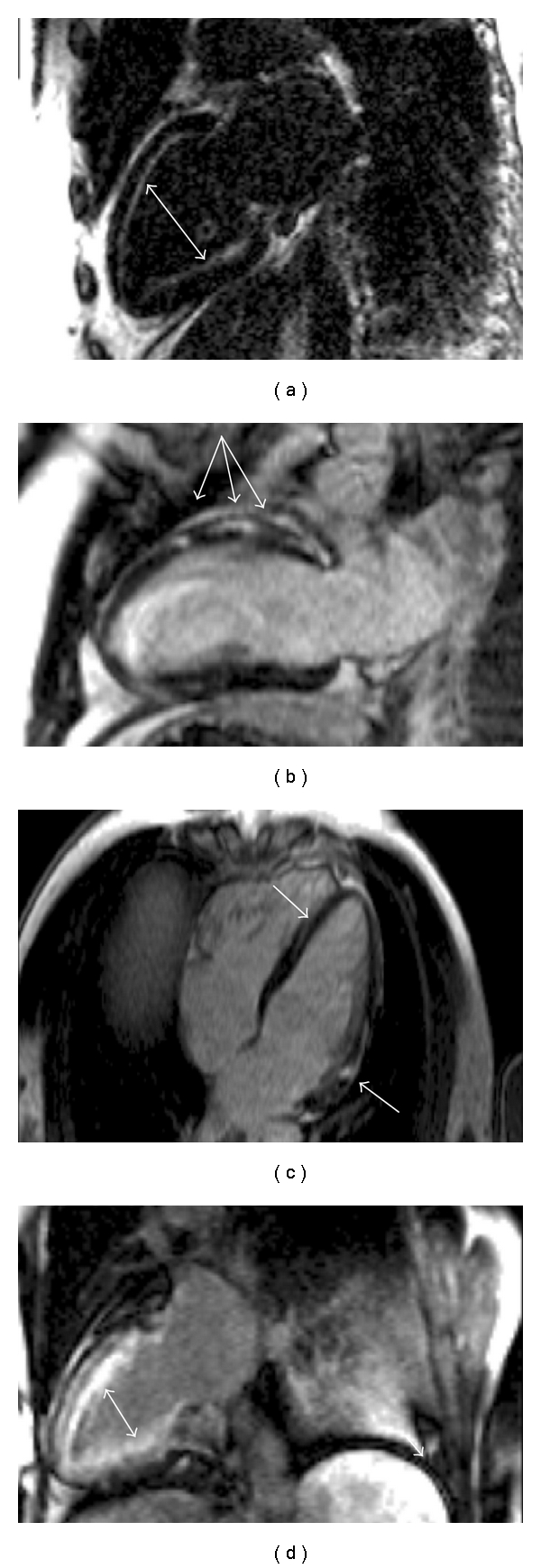
Gadolinium-enhanced imaging in specific cardiac diseases. (a) Subendocardial LGE in a patient with AL amyloidosis. (b) Mid anterior wall focal LGE lesions in a patient with sarcoidosis. (c) Epicardial and mid wall anterior LGE in a patient with myocarditis. (d) Subendocardial enhancement in a patient with Churgg Strauss syndrome.

**Table 1:** Sequences commonly employed during a CMR examination.

Sequence	When commonly used	Information obtained
Localisers	All studies	Allows cardiac piloting of all future images. Also allows extra cardiac structures to be assessed
Transverse Thoracic Stack	All studies	Assessment of extra cardiac thoracic structures
Cardiac long axis and LV short axis cines	All studies	Assessment of LV and RV volumes, systolic function and regional wall motion abnormality
Flow	Valve Disease/Shunts	Quantification of Qp : Qs shunt ratio. Quantification of valve regurgitant fraction and forward flow velocity.
T1 weighted turbo spin echo	ARVC assessment	Demonstration of myocardial fat
STIR	Acute Myocardial Infarction and Myocarditis	Demonstration of myocardial oedema
T2*	Iron overload syndromes (e.g., Beta Thalassaemia major)	Demonstration and quantification of myocardial and liver iron
Gadolinium First Pass Perfusion (with and without vasodilator stress)	Ischaemic Heart Disease, Cardiac mass assessment	Demonstration of perfusion defects in areas of ischaemic myocardium. Assessment of the vascularity of a cardiac mass
Gadolinium Enhanced Aortogram	Aortic Disease	Demonstration of dissection/dilatation of the aorta.
Early Gadolinium Inversion Recovery	Assessment of intracardiac mass	Demonstration of LV or left atrial appendage thrombus
Late Gadolinium Inversion Recovery	Nearly All Studies	Assessment of LV infarction and viability in ischaemic heart disease. Assessment of fibrosis in Cardiomyopathies.

## References

[1] http://www.heartstats.org/datapage.asp?id=752.

[2] http://www.ic.nhs.uk/webfiles/Services/NCASP/audits%20and%20reports/NHS_National_Heart_Failure_Audit_09_INTERACTIVE.pdf.

[3] Lloyd-Jones D, Adams RJ, Brown TM (2010). Executive summary: heart disease and stroke statistics-2010 update: a report from the american heart association. *Circulation*.

[4] Bruder O, Schneider S, Nothnagel D (2009). EuroCMR (European Cardiovascular Magnetic Resonance) registry. Results of the german pilot phase. *Journal of the American College of Cardiology*.

[5] Scott AD, Keegan J, Firmin DN (2011). Beat-to-beat respiratory motion correction with near 100% efficiency: a quantitative assessment using high-resolution coronary artery imaging. *Magnetic Resonance Imaging*.

[6] http://www.scmr.org/navigation/CMR-in-specific-circumstances.html.

[7] Kramer CM, Barkhausen J, Flamm SD, Kim RJ, Nagel E (2008). Standardized cardiovascular magnetic resonance imaging (CMR) protocols, society for cardiovascular magnetic resonance: board of trustees task force on standardized protocols. *Journal of Cardiovascular Magnetic Resonance*.

[8] Semelka RC, Tomei E, Wagner S (1990). Normal left ventricular dimensions and function: interstudy reproducibility of measurements with cine MR imaging. *Radiology*.

[9] Flett AS, Hasleton J, Cook C (2011). Evaluation of techniques for the quantification of myocardial scar of differing etiology using cardiac magnetic resonance. *Journal of the American College of Cardiology*.

[10] Mayr M, Burkhalter F, Bongartz G (2009). Nephrogenic systemic fibrosis: clinical spectrum of disease. *Journal of Magnetic Resonance Imaging*.

[11] Francis JM, Pennell DJ (2000). Treatment of claustrophobia for cardiovascular magnetic resonance: use and effectiveness of mild sedation. *Journal of Cardiovascular Magnetic Resonance*.

[12] Syed MA, Carlson K, Murphy M, Ingkanisorn WP, Rhoads KL, Arai AE (2006). Long-term safety of cardiac magnetic resonance imaging performed in the first few days after bare-metal stent implantation. *Journal of Magnetic Resonance Imaging*.

[13] Morrow DA, Antman EM, Charlesworth A (2000). TIMI risk score for ST-elevation myocardial infarction: a convenient, bedside, clinical score for risk assessment at presentation: an Intravenous nPA for Treatment of Infarcting Myocardium Early II trial substudy. *Circulation*.

[14] Stebbins A, Mehta RH, Armstrong PW (2010). A model for predicting mortality in acute st-segment elevation myocardial infarction treated with primary percutaneous coronary intervention: results from the assessment of pexelizumab in acute myocardial infarction trial. *Circulation*.

[15] Singh M, Holmes DR, Lennon RJ, Rihal CS (2010). Development and validation of risk adjustment models for long-term mortality and myocardial infarction following percutaneous coronary interventions. *Circulation*.

[16] Abdel-Aty H, Cocker M, Meek C, Tyberg JV, Friedrich MG (2009). Edema as a very early marker for acute myocardial ischemia: a cardiovascular magnetic resonance study. *Journal of the American College of Cardiology*.

[17] Anderson LJ, Holden S, Davis B (2001). Cardiovascular T2-star (T2*) magnetic resonance for the early diagnosis of myocardial iron overload. *European Heart Journal*.

[18] Ganame J, Messalli G, Dymarkowski S (2009). Impact of myocardial haemorrhage on left ventricular function and remodelling in patients with reperfused acute myocardial infarction. *European Heart Journal*.

[19] O’Regan DP, Ariff B, Neuwirth C, Tan Y, Durighel G, Cook SA (2010). Assessment of severe reperfusion injury with T2* cardiac MRI in patients with acute myocardial infarction. *Heart*.

[20] Mather AN, Fairbairn TA, Ball SG, Greenwood JP, Plein S (2011). Reperfusion haemorrhage as determined by cardiovascular MRI is a predictor of adverse left ventricular remodelling and markers of late arrhythmic risk. *Heart*.

[21] Bekkers SCAM, Smulders MW, Passos VL (2010). Clinical implications of microvascular obstruction and intramyocardial haemorrhage in acute myocardial infarction using cardiovascular magnetic resonance imaging. *European Radiology*.

[22] Bekkers SCAM, Yazdani SK, Virmani R, Waltenberger J (2010). Microvascular obstruction. Underlying pathophysiology and clinical diagnosis. *Journal of the American College of Cardiology*.

[23] Eitel I, Kubusch K, Strohm O Prognostic value and determinants of a hypointense infarct core in T_2_-weighted cardiac magnetic resonance in acute reperfused ST-elevation myocardial infarction.

[24] Reimer KA, Jennings RB (1979). The “wavefront phenomenon” of myocardial ischemic cell death. II. Transmural progression of necrosis within the framework of ischemic bed size (myocardium at risk) and collateral flow. *Laboratory Investigation*.

[25] Dall’Armellina E, Karamitsos TD, Neubauer S, Choudhury RP (2010). CMR for characterization of the myocardium in acute coronary syndromes. *Nature Reviews Cardiology*.

[26] Larose E, Rodés-Cabau J, Pibarot P (2010). Predicting late myocardial recovery and outcomes in the early hours of ST-segment elevation myocardial infarction. *Journal of the American College of Cardiology*.

[27] Kanderian AS, Renapurkar R, Flamm SD (2009). Myocardial viability and revascularization. *Heart Failure Clinics*.

[28] Kim RJ, Wu E, Rafael A (2000). The use of contrast-enhanced magnetic resonance imaging to identify reversible myocardial dysfunction. *The New England Journal of Medicine*.

[29] Mahrholdt H, Wagner A, Judd RM, Sechtem U (2002). Assessment of myocardial viability by cardiovascular magnetic resonance imaging. *European Heart Journal*.

[30] Beek AM, Kühl HP, Bondarenko O (2003). Delayed contrast-enhanced magnetic resonance imaging for the prediction of regional functional improvement after acute myocardial infarction. *Journal of the American College of Cardiology*.

[31] Maron MS, Olivotto I, Betocchi S (2003). Effect of left ventricular outflow tract obstruction on clinical outcome in hypertrophic cardiomyopathy. *The New England Journal of Medicine*.

[32] Moon JCC, Fisher NG, McKenna WJ, Pennell DJ (2004). Detection of apical hypertrophic cardiomyopathy by cardiovascular magnetic resonance in patients with non-diagnostic echocardiography. *Heart*.

[33] O’Hanlon R, Grasso A, Roughton M (2010). Prognostic significance of myocardial fibrosis in hypertrophic cardiomyopathy. *Journal of the American College of Cardiology*.

[34] Moon JCC, Sachdev B, Elkington AG (2003). Gadolinium enhanced cardiovascular magnetic resonance in Anderson-Fabry disease: evidence for a disease specific abnormality of the myocardial interstitium. *European Heart Journal*.

[35] Mestroni L, Maisch B, McKenna WJ (1999). Guidelines for the study of familial dilated cardiomyopathies. *European Heart Journal*.

[36] Assomull RG, Prasad SK, Lyne J (2006). Cardiovascular magnetic resonance, fibrosis, and prognosis in dilated cardiomyopathy. *Journal of the American College of Cardiology*.

[37] Basso C, Corrado D, Marcus FI, Nava A, Thiene G (2009). Arrhythmogenic right ventricular cardiomyopathy. *The Lancet*.

[38] Yilmaz A, Gdynia HJ, Baccouche H (2008). Cardiac involvement in patients with Becker muscular dystrophy: new diagnostic and pathophysiological insights by a CMR approach. *Journal of Cardiovascular Magnetic Resonance*.

[39] Marcus FI, McKenna WJ, Sherrill D (2010). Diagnosis of arrhythmogenic right ventricular cardiomyopathy/Dysplasia: proposed modification of the task force criteria. *Circulation*.

[40] Pfluger HB, Phrommintikul A, Mariani JA, Cherayath JG, Taylor AJ (2008). Utility of myocardial fibrosis and fatty infiltration detected by cardiac magnetic resonance imaging in the diagnosis of arrhythmogenic right ventricular dysplasia-a single centre experience. *Heart Lung and Circulation*.

[41] Chin TK, Perloff JK, Williams RG, Jue K, Mohrmann R (1990). Isolated noncompaction of left ventricular myocardium. A study of eight cases. *Circulation*.

[42] Jacquier A, Thuny F, Jop B (2010). Measurement of trabeculated left ventricular mass using cardiac magnetic resonance imaging in the diagnosis of left ventricular non-compaction. *European Heart Journal*.

[43] Baumgartner H, Bonhoeffer P, De Groot NMS (2010). ESC Guidelines for the management of grown-up congenital heart disease (new version 2010): the task force on the management of grown-up congenital heart disease of the European Society of Cardiology (ESC). *European Heart Journal*.

[44] Teo K, Disney P, Dundon B (2010). Assessment of atrial septal defects in adults comparing cardiovascular magnetic resonance with transoesophageal echocardiography. *Journal of Cardiovascular Magnetic Resonance*.

[45] Taylor AM (2008). Cardiac imaging: MR or CT? Which to use when. *Pediatric Radiology*.

[46] Maisch B, Seferović PM, Ristić AD (2004). Guidelines on the diagnosis and management of pericardial diseases executive summary; the task force on the diagnosis and management of pericardial diseases of the European society of cardiology. *European Heart Journal*.

[47] Ariyarajah V, Jassal DS, Kirkpatrick I, Kwong RY (2009). The utility of cardiovascular magnetic resonance in constrictive pericardial disease. *Cardiology in Review*.

[48] Masui T, Finck S, Higgins CB (1992). Constrictive pericarditis and restrictive cardiomyopathy: evaluation with MR imaging. *Radiology*.

[49] Soulen RL (1991). Magnetic resonance imaging of great vessel, myocardial, and pericardial disease. *Circulation*.

[50] Vahanian A, Baumgartner H, Bax J (2007). Guidelines on the management of valvular heart disease: the task force on the management of valvular heart disease of the European society of cardiology. *European Heart Journal*.

[51] Bonow RO, Carabello BA, Chatterjee K (2006). ACC/AHA 2006 guidelines for the management of patients with valvular heart disease: a report of the American College of Cardiology/American Heart Association Task Force on Practice Guidelines (Writing Committee to Revise the 1998 Guidelines for the Management of Patients with Valvular Heart Disease)—developed in collaboration with the Society of Cardiovascular Anesthesiologists. *Circulation*.

[52] Westenberg JJM, Doornbos J, Versteegh MIM (2005). Accurate quantitation of regurgitant volume with MRI in patients selected for mitral valve repair. *European Journal of Cardio-thoracic Surgery*.

[53] Caruthers SD, Lin SJ, Brown P (2003). Practical value of cardiac magnetic resonance imaging for clinical quantification of aortic valve stenosis: comparison with echocardiography. *Circulation*.

[54] Kim JS, Judson MA, Donnino R (2009). Cardiac sarcoidosis. *American Heart Journal*.

[55] Nunes H, Freynet O, Naggara N (2010). Cardiac sarcoidosis. *Seminars in Respiratory and Critical Care Medicine*.

[56] Cheong BYC, Muthupillai R, Nemeth M (2009). The utility of delayed-enhancement magnetic resonance imaging for identifying nonischemic myocardial fibrosis in asymptomatic patients with biopsy-proven systemic sarcoidosis. *Sarcoidosis Vasculitis and Diffuse Lung Diseases*.

[57] Liu PP, Mason JW (2001). Advances in the understanding of myocarditis. *Circulation*.

[58] Kawai C (1999). From myocarditis to cardiomyopathy: mechanisms of inflammation and cell death: learning from the past for the future. *Circulation*.

[59] Friedrich MG, Sechtem U, Schulz-Menger J (2009). Cardiovascular magnetic resonance in myocarditis: a JACC white paper. *Journal of the American College of Cardiology*.

[60] Leurent G, Langella B, Fougerou C (2011). Diagnostic contributions of cardiac magnetic resonance imaging in patients presenting with elevated troponin, acute chest pain syndrome and unobstructed coronary arteries. *Archives of Cardiovascular Diseases*.

[61] Modell B, Khan M, Darlison M (2000). Survival in *β*-thalassaemia major in the UK: data from the UK thalassaesnia register. *The Lancet*.

[62] Maceira AM, Joshi J, Prasad SK (2005). Cardiovascular magnetic resonance in cardiac amyloidosis. *Circulation*.

[63] Kholová I, Niessen HWM (2005). Amyloid in the cardiovascular system: a review. *Journal of Clinical Pathology*.

[64] Alvarez-Sala R, Prados C, Armada E, Del Arco A, Villamor J (1995). Congestive cardiomyopathy and endobronchial granulomas as manifestations of Churg-Strauss syndrome. *Postgraduate Medical Journal*.

[65] Wassmuth R, Göbel U, Natusch A (2008). Cardiovascular magnetic resonance imaging detects cardiac involvement in churg-strauss syndrome. *Journal of Cardiac Failure*.

[66] World Health Organisation (2002). *The World Health Report 2002. Annexes and Tables*.

[67] Rochitte CE, Oliveira PF, Andrade JM (2005). Myocardial delayed enhancement by magnetic resonance imaging in patients with Chagas’ disease: a marker of disease severity. *Journal of the American College of Cardiology*.

[68] Epstein AE, DiMarco JP, Ellenbogen KA (2008). ACC/AHA/ HRS 2008 guidelines for device based therapy of cardiac rhythm abnormalities: a report of the American College of Cardiology / American Heart Association task force on practice guidelines (writing committee to revise the ACC/AHA/NAPSE 2002 guideline update for implantation of cardiac pacemakers and antiarrhythmia devices) developed in collaboration with the American Association for Thoracic Surgery and Society of Thoracic Surgeons. *Journal of the American College of Cardiology*.

[69] Chung ES, Leon AR, Tavazzi L (2008). Results of the predictors of response to crt (prospect) trial. *Circulation*.

[70] Leyva F (2010). Cardiac Resynchronisation therpay guided by cardiovascular magnetic resonance. *Journal of Cardiovascular Magnetic Resonance*.

[71] White JA, Yee R, Yuan X (2006). Delayed enhancement magnetic resonance imaging predicts response to cardiac resynchronisation therapy in patients with intraventricular dyssynchrony. *Journal of the American College of Cardiology*.

[72] Chiribiri A, Kelle S, Gotze S (2008). Visualisation of the cardiac venous system using cardiac magnetic resonance. *American Journal of Cardiology*.

[73] Piechnik SK, Ferreira VM, Dall'Armellina E (2010). Shortened Modified Look-Locker Inversion recovery (ShMOLLI) for clinical myocardial T1-mapping at 1.5 and 3 T within a 9 heartbeat breathhold. *Journal of Cardiovascular Magnetic Resonance*.

[74] Flett AS, Hayward MP, Ashworth MT (2010). Equilibrium contrast cardiovascular magnetic resonance for the measurement of diffuse myocardial fibrosis: preliminary validation in humans. *Circulation*.

